# Bioabsorbable Hydrogel Coating for Infection Prevention in Fracture Fixation: A Retrospective Matched Case–Control Study

**DOI:** 10.3390/ph19030518

**Published:** 2026-03-23

**Authors:** Carlo Ciccullo, Marco Grassi, Marco Antonio Carletti, Claudia Bevilacqua, Danilo Francesco Chirillo, Simone Domenico Aspriello, Antonio Pompilio Gigante

**Affiliations:** 1 Clinical Orthopedics, Department of Clinical and Molecular Sciences, Università Politecnica delle Marche Azienda Ospedaliera Universitaria delle Marche, Via Conca 71, 60126 Ancona, Italy; marco.grassi7@gmail.com (M.G.); m.antonio.carletti@gmail.com (M.A.C.); claudia.bevilacqua@ospedaliriuniti.marche.it (C.B.); a.gigante@univpm.it (A.P.G.); 2 Azienda Ospedaliero-Universitaria SS. Antonio e Biagio e Cesare Arrigo, Via Venezia 16, 15121 Alessandria, Italy; dchirillo@aslal.al; 3 ASD Dental Clinic, 61121 Pesaro, Italy; simonedomenico@yahoo.it; 4 IRCCS INRCA, Via Della Montagnola, 60127 Ancona, Italy

**Keywords:** hydrogel, DAC, infections, hospital-acquired infections, trauma surgery

## Abstract

**Background/Objectives**: Hospital-acquired and fracture-related infections remain major complications in orthopedic trauma surgery, with significant clinical and socio-economic impact. Antibacterial implant surface coatings represent a promising strategy to reduce early postoperative bacterial adhesion and biofilm formation. **Methods**: This retrospective matched case–control study evaluated the clinical effectiveness of an antibiotic-free fast-resorbable hyaluronic acid and poly-d, l-lactide hydrogel (DAC^®^) applied intraoperatively to orthopedic implants. A total of 222 patients with comorbidities who underwent open reduction and internal fixation between May 2023 and April 2024 in two trauma centers were included: 99 patients received the DAC^®^ coating and 123 served as controls with standard fixation. The primary endpoint was infection incidence within 6 months; secondary endpoints included wound complications, revision surgery, prolonged antibiotic therapy, and bone healing. **Results**: Postoperative infection incidence was significantly lower in the DAC^®^ group compared with controls (0.7% vs. 5.3%; *p* = 0.0363). Wound complications were also reduced (1.3% vs. 8.0%; *p* = 0.028), and only one patient in the DAC^®^ cohort required additional surgical interventions or prolonged antibiotic therapy. Bone healing outcomes were comparable between groups, with no delayed unions reported in the treated cohort. **Conclusions**: Even if larger prospective studies with longer follow-up are required to further confirm these findings and better define long-term safety and effectiveness, the routine intraoperative use of DAC^®^ hydrogel without antibiotic loading appears to be a safe and promising strategy to reduce early postoperative infections and wound complications in orthopedic trauma patients with comorbidities.

## 1. Introduction

Hospital-acquired infections (HAIs) and infections after fracture fixation (IAFFs) in orthopedic surgery continue to represent a significant burden on healthcare systems worldwide due to increasing rates of antibiotic resistance. Furthermore, IAFFs have been known to result in non-union, loss of function and even amputation. They can be a source of concern both in terms of morbidity and mortality and also represent a significant socio-economic burden [1,2,3]. Postoperative infections following fracture fixation are among the most severe complications in orthopedic trauma care. A success rate of between 70% and 90% has been shown for treating IAFF. Some studies report an incidence of IAFF in closed fractures of between 1% and 2%. According to recent studies, the incidence of IAFF in open fractures is around 30% [4,5]. It is estimated that nosocomial infections are responsible for approximately 16 million additional hospital days each year in Europe, adding billions of euros to the cost of the healthcare system. Furthermore, the prevalence of these infections has increased by 0.06% per year, with the highest rates observed in low-income regions [6,7,8]. According to some estimates, 11 out of every 100 surgical patients may develop a surgical site infection within 30 days of undergoing surgery [9]. Such infections have a significant impact on patient outcomes and on the efficiency of healthcare systems in trauma and orthopedic surgery [10]. Current research focuses on best diagnosis methods [9,11] and on adopting new preventive treatments to avoid surgical site infection and the formation of bacterial biofilms on synthetic materials in patients undergoing fracture reduction and synthesis surgery.

In this context, antibacterial coatings on implants may represent a promising solution to reduce post-surgical infections [12]. At a recent international consensus meeting on peri-prosthetic joint infections, a strong recommendation was made on the need to develop effective antibacterial surfaces that prevent bacterial adhesion and colonization of implants and proliferation in surrounding tissues [13]. However, only a limited number of antibacterial coating technologies are currently available in orthopedics and traumatology [14]. Recently, a fast-resorbable hydrogel coating has been developed that can be loaded intraoperatively. The observations recorded indicated that bacterial colonization occurs during the initial hours following the implantation process.

Biofilms are mainly composed of bacterial colonies and extracellular polymeric substances, such as polysaccharides, proteins, and DNA. They have an extremely complex formation mechanism. In general, biofilm formation can be divided into five stages: (1) reversible bacterial adhesion; (2) irreversible adhesion; (3) initial biofilm formation; (4) biofilm maturation; and (5) the release of toxins and free bacteria to initiate a new cycle of biofilm formation [15,16,17,18].

Furthermore, it was established that systemic prophylactic measures administered for a limited duration were as efficacious as those implemented over an extended timeframe in the prevention of post-surgical infections. This pioneering coating technology thus introduced the concept of “short-term local protection” for the implant [19].

Recent findings have indicated that a hydrogel with a defensive antibacterial coating (DAC^®^) has proven to be a promising innovation in the field of osteosynthesis-related infections. It is particularly effective across a range of patient groups. The composition of the bioresorbable hydrogel under scrutiny consists of two polymers: sodium hyaluronate (hyaluronic acid, 85%) and poly-d, l-lactide (15%). The amalgamation of these elements results in the formation of a temporary absorbable barrier on implant surfaces, reducing bacterial adhesion without impairing bone healing. This coating can be applied to metal orthopedic implants and soft tissues during surgical procedures [20,21,22,23,24]. The present study aims to evaluate the clinical effectiveness of DAC^®^ hydrogel without antibiotic for the intervention in reducing occupational accidental injuries (OAIs) within our department. The implants studied include plates, screws, and intramedullary nails used for fracture stabilization.

## 2. Results

Both cohorts were comparable with respect to demographic variables and perioperative characteristics. Most fractures were closed injuries involving the lower extremities, most commonly the femur, tibia, and ankle. The incidence of postoperative infection was significantly lower in the DAC^®^ group, with one case (0.7%), compared with eight cases (5.3%) in the control group (*p* = 0.0363); odds ratio of 0.15 (95% CI, 0.02–1.19). The mean time to infection onset was 35 days in the DAC^®^ group and 36 ± 5 days in the control group. Methicillin-resistant *Staphylococcus aureus* (MRSA) was identified in five of the eight infections observed in the control group. Wound-related complications occurred in 1.3% of patients in the DAC^®^ group and in 8.0% of control patients (OR, 0.12; 95% CI, 0.01–0.92), demonstrating a statistically significant difference (*p* = 0.028). Regarding reinterventions and antibiotic management, two patients in the control group required debridement, antibiotics, and implant retention (DAIR), followed by implant removal, and nine patients required prolonged antibiotic therapy combined with chlorhexidine wound irrigation; no such interventions were necessary in the DAC^®^ group (OR of 0.061; 95% CI, 0.004–1.05; *p* = 0.0023). At the 6-month follow-up, all infections had resolved except for one persistent case in the control group. Our clinical and radiographic follow-up data indicate that the DAC^®^ hydrogel coating does not interfere with bone healing. Delayed bone union was observed in two control patients, whereas no cases of delayed healing were reported in the DAC^®^ cohort.

## 3. Discussion

Hydrophilic hydrogels based on biopolymers can be used either alone or as carriers in combination with antibiotics. These coatings act by preventing bacterial adhesion to implant surfaces, thereby inhibiting biofilm formation. Hydrophilic hyaluronic acid (HA)-based hydrogel coatings have attracted particular attention from both experimental and clinical perspectives. Such coatings transform implant surfaces into hydrophilic substrates, which have been shown to reduce bacterial adhesion, especially during the early postoperative period [25,26,27].

Moreover, hydrogel-based coatings can provide a physical barrier against bacterial colonization when loaded with antibacterial agents [28,29]. Some authors have also suggested that biopolymers such as hyaluronic acid may counteract the effects of hyaluronidase, an enzyme produced by many pathogens to facilitate penetration of host defenses. The bacteriostatic properties of HA have been demonstrated experimentally and in various surgical contexts, including maxillofacial and dental surgery [30,31]. Furthermore, the association of HA with PLGA particles could improve the stability of HA, reduce the in situ inflammation, and prolong the residence time of HA in the intra-articular compartment [32].

DAC^®^ represents the only commercially available biopolymer-based (hyaluronic acid) coating specifically designed to protect implant surfaces from bacterial adhesion and biofilm formation. Several studies have indicated that application of DAC^®^ gel can provide local protection for joint implants in selected populations, particularly patients with comorbidities or a high risk of infection, potentially resulting in substantial cost savings.

It has been shown to be a promising strategy for preventing implant-related infections in orthopedic trauma surgery [33]. A multicenter randomized controlled trial was conducted, involving a total of 256 patients who had undergone internal fixation for closed fractures, with the application of DAC^®^. The study’s authors, Malizos et al. [34], concluded that there was a significant reduction in surgical site infections (SSIs). Similarly, De Meo et al. [25] retrospectively evaluated the effectiveness of DAC^®^ in preventing infections in 27 high-risk patients, concluding that local prophylaxis with DAC^®^ effectively reduced infection incidence compared with the predicted preoperative risk.

In the present study, the two groups were comparable in terms of sample size and baseline characteristics (Table 1). The distribution by gender was well balanced, with a slight predominance of females in the control group (52.0%) and a slight predominance of males in the DAC group (54%), with no statistically significant differences. The mean age was marginally higher in the control group (62.5 ± 19.5 years) than in the DAC group (58.7 ± 18.6 years). However, the substantial overlap in standard deviations suggests that there are no clinically or statistically significant differences. We also demonstrate that there were no statistically significant discrepancies between the two groups with regard to sample size and initial demographics. The distribution by gender was well balanced, with a slight predominance of females in the control group (52.0%) and a slight predominance of males in the DAC group (54%), with no statistically significant differences. The mean age was marginally higher in the control group (62.5 ± 19.5 years) than in the DAC group (58.7 ± 18.6 years). However, the substantial overlap in standard deviations suggests that there are no clinically or statistically significant differences. The distribution of cardiovascular comorbidities, encompassing general cardiovascular disease and atrial fibrillation, was found to be uniform across the study groups. A similar uniformity was observed in the distribution of smoking, obesity, lung disease/COPD, neoplasms, allergies, osteoporosis, autoimmune diseases, chronic kidney disease, gastrointestinal and liver disorders, orthopedic and thyroid diseases, and psychiatric or neurodegenerative conditions, with no statistically significant differences observed.

A second statistically significant difference was observed concerning dyslipidaemia, which was present in the control group (4.9%) but absent in the DAC group (*p* = 0.035). Despite the low overall prevalence, this disparity must be considered when interpreting the study’s findings. Previous surgeries and the presence of other comorbidities were more prevalent in the control group; however, these differences did not reach statistical significance.

The demographic variables, fracture characteristics, surgical treatment, and follow-up results are summarized in Table 2. The presence of at least one risk factor or comorbidity was comparable between the groups (68.3% in the control group vs. 66.7% in the DAC group). The mean number of comorbidities per patient was marginally higher in the control group (2.0 ± 1.8) than in the DAC group (1.7 ± 1.7), although this difference was not statistically significant. In addition, the distribution of patients according to the total number of comorbidities (0, 1–2 or >2) was found to be largely comparable between the two groups.

About anatomical distribution, lower limb fractures were observed to be more prevalent in both groups, with a marginally higher percentage recorded in the control group (75.6% versus 67.7%). Conversely, upper limb involvement was more prevalent in the DAC group (32.3% versus 24.4%); however, these differences did not attain statistical significance.

In relation to surgical management, the most prevalent surgical intervention employed was the use of plates and screws, with 78.0% of cases in the control group and 80.8% in the DAC group undergoing this treatment. Intramedullary nailing was observed to be more prevalent in the control group than in the DAC group (18.7% vs. 8.1%), while alternative fixation techniques were utilized with greater frequency in the DAC group (11.1% vs. 3.3%). Clear and clinically relevant differences emerged during follow-up, particularly in the context of early wound healing. A statistically significant difference was observed at the two-week mark, with a higher rate of “non-healing” wounds in the control group than in the DAC group (28.5% vs. 6.1%, *p* = 0.001). This discrepancy was also observed in the control group one month after surgery, where delayed wound healing was significantly more prevalent (15.4% vs. 3.0%, *p* = 0.003).

However, subsequent follow-ups at 3 and 6 months revealed comparable wound conditions between the groups, with high rates of satisfactory healing. Our results are consistent with the literature currently available. The follow-up period was limited to 6 months because beyond this time frame, in the presence of septic malunion or failure of union, a surgical revision procedure would have been necessary. This would have rendered further follow-up irrelevant for this study’s objectives.

As reported by Romanò et al. [21], a significant reduction in infections was observed in the group of patients treated with DAC^®^ loaded with antibiotics in comparison to the standard control treatment.

Three months after treatment, radiographic and clinical healing was slightly more frequent in the DAC group (89.9% versus 83.7%), although this difference was not statistically significant. The incidence of non-union at three months was low in both groups (1.6% in the control group versus 1.0% in the DAC group).

By 6 months, almost all patients in both groups had achieved clinical and radiographic healing. Only one patient in the DAC group (1.0%) required revision surgery, compared to none in the control group. There was a statistically significant difference in infectious complications in favor of the DAC group. Superficial infections were significantly more prevalent in the control group (12.2%) than in the DAC group (2.0%) (*p* = 0.005). The most identified pathogens were Staphylococcus aureus and MRSA; in several cases, no pathogen was isolated. Deep infections were observed exclusively in the control group (4.1%), with none occurring in the DAC group. Although this difference did not reach statistical significance, it suggests a clinically relevant trend. Similarly, antibiotic therapy was only necessary for patients in the control group (4.1%).

To the best of our knowledge, this is the first study on the efficacy and safety of DAC coating without the addition of antibiotic for internal osteosynthesis.

Our findings confirm that DAC^®^—composed of hyaluronic acid and polylactic acid—creates a temporary physical barrier that modifies implant surface properties, reducing bacterial colonization during the critical early postoperative period. Based on the manufacturer’s data and current literature, the coating is fully absorbed within 72–96 h and shows excellent biocompatibility [35,36,37]. Our findings align with this: we observed no interference with bone healing, as evidenced by both radiographic imaging and clinical follow-up throughout the study. Furthermore, fewer wound complications were observed, resulting in reduced need for prolonged antibiotic therapy.

Nevertheless, this study has limitations: a potential limitation of the present study, in fact, is the relatively small number of infection events, which may reduce the statistical power and limit the ability to detect small differences between groups. Consequently, the findings should be interpreted with caution, and larger prospective studies with larger cohorts and longer observation periods are warranted to further validate these findings and to evaluate long-term safety and efficacy. Another imitation of this study is the relatively small number of open fractures, which are known to carry a higher risk of postoperative infection. Future studies should specifically evaluate the efficacy of DAC^®^ hydrogel without the addition of antibiotics in this high-risk subgroup to provide more robust evidence on its preventive potential.

## 4. Materials and Methods

A retrospective matched case–control study was conducted in two trauma centers: Azienda Ospedaliera Universitaria delle Marche (AOU Marche) and Azienda Ospedaliera Uniersaitaria di Alessandria (AOU Alessandria). Patients with one or more comorbidities who underwent ORIF (Open Reduction and Internal Fixation) between May 2023 and April 2024 were included. The choice to apply the DAC^®^ coating was made intraoperatively, weighing the patient’s overall health and the complexity of the fracture against the risk of postoperative infection. The DAC^®^ group (n = 99) received the hydrogel coating on implants; controls (n = 123) underwent standard fixation without coating. A total of 89 patients received treatment at the AOU Marche. Of these patients, 54 were treated with DAC and 35 were not treated with DAC. A total of 133 patients received treatment at AOU Alessandria, 45 of whom were treated with DAC and 88 without. The patients were evaluated with standard clinical and radiographic follow-up at 1, 3, and 6 months. The evaluation of patients and collection of their medical history data were delegated to two independent doctors, one in each hospital. Matching criteria included age, sex, fracture type, and comorbidity profile. All patients received systemic antibiotic prophylaxis according to institutional protocols based on the national guidelines established by the Italian Society of Orthopaedics and Traumatology (SIOT) [21,38]. Primary endpoint: Incidence of OAIs within 6 months. Secondary endpoints: Wound complications, revision surgery, prolonged antibiotic therapy, and bone healing.

Matching between the DAC^®^ group and the control group was performed using a frequency-matching approach based on age, sex, fracture type (upper vs. lower limb, open vs. closed), and comorbidity profile, in order to ensure comparable baseline characteristics between the two cohorts. Patients in the control group were selected from the same study period and institutions in order to minimize potential selection bias.

Due to the retrospective observational design of this study, a formal a priori sample size calculation or power analysis was not performed.

Statistical analysis was performed using R Statistical Software (version 4.0.0; R Foundation for Statistical Computing). Continuous variables are expressed as the mean ± standard deviation (SD) and medians and first and third quartiles [Q1–Q3]. The Shapiro–Wilk normality test was used to evaluate the normal distribution of the sample. The between-group differences for continuous variables were evaluated with the unpaired Student’s *t* test or Mann–Whitney test, according to the characteristics of the data distribution. Categorical variables are expressed in numbers of cases and frequencies; their differences were tested using the chi-square test or Fisher’s exact test, as appropriate. For all analyses, the significance was set at *p* < 0.05. All patients signed informed consent to participate in the study and to allow the use of clinical data for research purposes. The study was approved by the Internal Review Board of the Polytechnic University of Marche (ID 1825 n.90/2021—approved on 5 March 2021), Ancona, Italy. The study was conducted in accordance with the ethical principles outlined in the Declaration of Helsinki.

### Surgical Procedure and DAC Preparation

**Figure 1:**
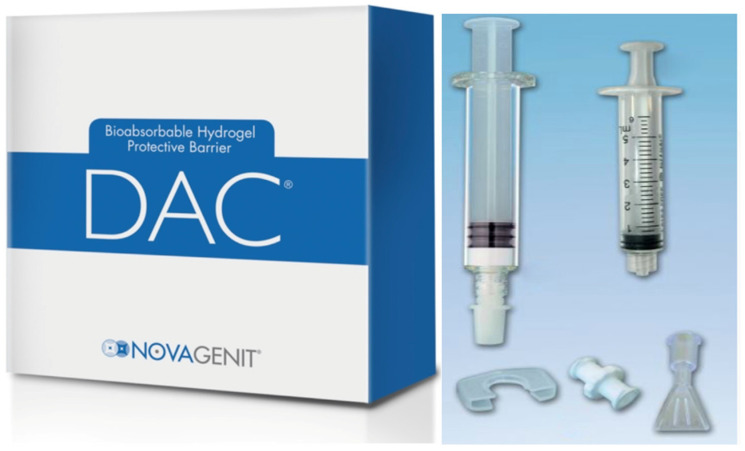
The image shows the DAC kit, box, and sterile contents that were used to conduct this study.

Following a standard preoperative physical examination, all patients were treated in accordance with contemporary principles of fracture reduction and internal osteosynthesis. The selection of the surgical approach and the method of osteosynthesis were delegated to the discretion of each participating surgeon. Systemic antibiotic prophylaxis was administered in accordance with the perioperative protocol, which stipulated the administration of a single dose of the antibiotic of choice at each center [38]. All patients administered low-weight heparin for the purpose of deep vein thrombosis prophylaxis. This treatment was initiated on the day of surgery and continued for a period of 4–6 weeks postoperatively. The DAC^®^ (Defensive Antibacterial Coating—Novagenit S.r.l., Mezzolombardo, Italy) hydrogel (Figure 1) was reconstituted in strict accordance with the manufacturer’s instructions. In summary, the pre-filled syringe containing 300 mg of sterile DAC powder was filled with a solution of 5 mL of sterile water for injections during surgery. The solution was prepared without the addition of antibiotics. Subsequently, the product was meticulously applied to the synthesis material using a specialized brush provided by the manufacturers. Following this step, the incision was meticulously closed with absorbable sutures.

## 5. Conclusions

The routine intraoperative use of DAC^®^ hydrogel without antibiotic loading was associated with a reduction in occupational accidental injuries and fracture-related infections in patients with comorbidities. The intervention was well tolerated and did not appear to adversely affect bone healing. Nevertheless, these findings should be interpreted with caution, and further large-scale, prospective studies with extended follow-up are required to confirm the observed effects and to determine the potential role of this strategy within established infection prevention protocols.

## Figures and Tables

**Table 1:** Demographics.

	CONTROL		DAC		*p*
	*n*	%	*n*	%	
SIMPLE SIZE	123	100.0	99	100	
SEX					
F	64	52.0	46	46	
M	59	48.0	53	54	
AGE					
Mean	62.5		58.7		
St. Dev	19.5		18.6		
RISK FACTORS					
DIABETES					
No	116	94.3	93	93.9	
Yes	7	5.7	6	6.1	
CORTICOSTEROIDS					
No	116	94.3	99	100	*p =* 0.018
Yes	7	5.7	0	0	
HIGH BLOOD PRESSURE					
No	76	61.8	65	65.7	
Yes	47	38.2	34	34.3	
HEART DISEASE					
No	97	78.9	76	76.8	
Yes	26	21.1	23	23.2	
FA (ATRIAL FIBRILLATION)					
No	113	91.9	93	93.9	
Yes	10	8.1	6	6.1	
SMOKE					
No	115	93.5	94	94.9	
Yes	8	6.5	5	5.1	
OBESITY					
No	119	96.7	95	96.0	
Yes	4	3.3	4	4.0	
LUNG DISEASES/BPCO					
No	115	93.5	97	98.0	
Yes	8	6.5	2	2.0	
TUMORS					
No	114	92.7	90	90.9	
Yes	9	7.3	9	9.1	
ALLERGY					
No	119	96.7	97	98.0	
Yes	4	3.3	2	2.0	
OSTEOPOROSIS					
No	117	95.1	93	93.9	
Yes	6	4.9	6	6.1	
AUTOIMMUNE DISEASES					
No	118	95.9	95	96.0	
Yes	5	4.1	4	4.0	
CKD (CHRONIC KIDNEY DISEASE)					
No	116	94.3	97	98.0	
Yes	7	5.7	2	2.0	
GASTROINTESTINAL DISEASES					
No	113	91.9	94	94.9	
Yes	10	8.1	5	5.1	
LIVER DISEASES					
No	116	94.3	92	92.9	
Yes	7	5.7	7	7.1	
ORTHOPEDIC DISEASES					
No	114	92.7	91	91.9	
Yes	9	7.3	8	8.1	
THYROID DISEASES					
No	116	94.3	95	96.0	
Yes	7	5.7	4	4.0	
DYSLIPIDEMIA					
No	117	95.1	99	100.0	*p* = 0.035
Yes	6	4.9	0	0	
PSYCHIATRIC DISEASES					
No	113	91.9	94	94.9	
Yes	10	8.1	5	5.1	
PREVIOUS SURGEONS					
No	75	61.0	69	69.7	
Yes	48	39.0	30	30.3	
OTHER PATHOLOGY					
No	97	78.9	85	85.9	
Yes	26	21.1	14	14.1	

**Table 2:** Demographics and follow-up.

	CONTROL		DAC		*p*
	*n*	%	*n*	%	
RISK FACTORS					
No	39	31.7	33	33.3	
Yes	84	68.3	66	66.7	
MEAN NUMBER OF COMORBIDITIES					
Mean	2.0		1.7		
St. Dev	1.8		1.7		
TOTAL NUMBER OF COMORBIDITIES					
0	33	26.8	33	33.3	
1–2	45	36.6	36	36.4	
>2	45	36.6	30	30.3	
ANATOMICAL REGION					
Upper Limb	30	24.4	32	32.3	
Lower Limb	93	75.6	67	67.7	
OPEN FRACTURES					
No	111	90.2	93	93.9	
Yes	12	9.8	6	6.1	
COMMINUTED FRACTURES					
No	113	91.9	91	91.9	
Yes	10	8.1	8	8.1	
OSTEOSYNTHESIS					
Intramid. Nail	23	18.7	8	8.1	
Plate and Screws	96	78.0	80	80.8	
Others	4	3.3	11	11.1	
FOLLOW-UP					
WOUND HEALING AT 2 WEEKS					
No	35	28.5	6	6.1	*p* = 0.001
Yes	88	71.5	93	93.9	
WOUND HEALING AT 1 MONTH					
No	19	15.4	3	3.0	*p* = 0.003
Yes	104	84.6	96	97.0	
WOUND HEALING AT 3 MONTHS					
No	7	5.7	3	3.0	
Yes	116	94.3	96	97.0	
WOUND HEALING AT 6 MONTHS					
No	8	6.5	6	6.1	
Yes	115	93.5	93	93.9	
CLINICAL (X-RAY) HEALING AT 3 MONTHS					
No	20	16.3	10	10.1	
Yes	103	83.7	89	89.9	
NONUNION					
No	121	98.4	98	99.0	
Yes	2	1.6	1	1.0	
CLINICAL (X-RAY) HEALING AT 6 MONTHS					
No	0	0.0	1	1.0	
Yes	123	100.0	98	99.0	
REVISION SURGERY					
No	123	100.0	98	99.0	
Yes	0	0.0	1	1.0	
SUPERFICIAL INFECTIONS					
No	108	87.8	97	98.0	*p* = 0.005
Yes	15	12.2	2	2.0	
PATHOGEN					
Enterococco	3	2.4			
MRSA	1	0.8			
*S. Aureus*	3	2.4	2	2.0	
*S. Aureus* Coagulasi Neg.	1	0.8			
N.I.	6	4.9			
DEEP INFECTIONS					
No	118	95.9	99	100	
Yes	5	4.1	0	0	
PATHOGEN					
MRSA	2	1.6			
*S. Aureus*	2	1.6			
N.I.	1	0.8			
ANTIBIOTIC THERAPY					
No	118	95.9	99	100	
Yes	5	4.1			

## Data Availability

The original contributions presented in this study are included in the article. Further inquiries can be directed to the corresponding author.
